# Social and health factors associated with adverse treatment outcomes among people with multidrug-resistant tuberculosis in Sierra Leone: a national, retrospective cohort study

**DOI:** 10.1016/S2214-109X(22)00004-3

**Published:** 2022-03-15

**Authors:** Rashidatu Fouad Kamara, Matthew J Saunders, Foday Sahr, Juan E Losa-Garcia, Lynda Foray, Geraint Davies, Tom Wingfield

**Affiliations:** a Ministry of Health and Sanitation, Sierra Leone; b Rey Juan Carlos University and Hospital Universitario Fundación Alcorcon, Madrid, Spain; c National Tuberculosis Programme, Sierra Leone; d Faculty of Public Health and Policy, London School of Hygiene & Tropical Medicine, London, UK; e Department of Microbiology, University of Sierra Leone, Sierra Leone; f Department of Clinical Infection, Microbiology & Immunology, University of Liverpool, Liverpool, UK; g Departments of International Public Health and Clinical Sciences, Liverpool School of Tropical Medicine, Liverpool, UK; h Tropical and Infectious Disease Unit, Liverpool University Hospitals NHS Foundation Trust, Liverpool, UK; i World Health Organization Collaborating Centre for Tuberculosis and Social Medicine, Department of Global Public Health, Karolinska Institutet, Stockholm, Sweden

## Abstract

**Background:**

Multidrug-resistant tuberculosis (MDR-TB) is a global health emergency. We aimed to evaluate treatment outcomes among people with MDR-TB in Sierra Leone and investigate social and health factors associated with adverse treatment outcomes.

**Methods:**

This national, retrospective cohort study recruited all people notified with MDR-TB to the Sierra Leone National TB Programme, admitted to Lakka hospital (Lakka, Western Area Rural District, Freetown, Sierra Leone) between April, 2017, and September, 2019. Participants were followed up to May, 2021. People who were eligible but had no social or health data available, or were subsequently found to have been misdiagnosed, were excluded from participation. MDR-TB treatment was with the 2017 WHO-recommended short (9–11 month) or long (18–24 month) aminoglycoside-containing regimens. Multivariable logistic regression models examined associations of programmatic social and health data with WHO-defined adverse treatment outcomes (death, treatment failure, loss to follow-up).

**Findings:**

Of 370 notified MDR-TB cases, 365 (99%) were eligible for study participation (five participants were excluded due to lack of social or health data or misdiagnosis). Treatment was started by 341 (93%) of 365 participants (317 received the short regimen, 24 received the long regimen, and 24 received no treatment). Median age was 35 years (IQR 26–45), 263 (72%) of 365 were male and 102 (28%) were female, 71 (19%) were HIV-positive, and 127 (35%) were severely underweight (body-mass index <16·5 kg/m^2^). Overall, 267 (73%) of 365 participants had treatment success, 95 (26%) had an adverse outcome, and three (1%) were still on treatment in May, 2021. Age 45–64 years (adjusted odds ratio [aOR] 2·4, 95% CI 1·2–5·0), severe underweight (aOR 4·2, 1·9–9·3), untreated HIV (aOR 10, 2·6–40·0), chronic lung disease (aOR 2·0, 1·0–4·2), previously unsuccessful drug-sensitive tuberculosis retreatment (aOR 4·3, 1·0–19), and a long regimen (aOR 6·5, 2·3–18·0) were associated with adverse outcomes. A sensitivity analysis showed that prothionamide resistance (aOR 3·1, 95% CI 1·5–10·0) and aminoglycoside-related complete deafness (aOR 6·6, 1·3–35) were independently associated with adverse outcomes.

**Interpretation:**

MDR-TB treatment success in Sierra Leone approached WHO targets and the short regimen was associated with higher success. The social and health factors associated with adverse outcomes in this study suggest a role for integrated tuberculosis, HIV, and non-communicable disease services alongside nutritional and socioeconomic support for people with MDR-TB and emphasise the urgent need to scale up coverage of all-oral aminoglycoside-sparing regimens.

**Funding:**

Wellcome Trust, Joint Global Health Trials.

## Introduction

Multidrug-resistant tuberculosis (MDR-TB), defined as resistance to both rifampicin and isoniazid, remains a major public health concern, especially in low-income and middle-income countries. In 2019, about half a million new cases of rifampicin-resistant tuberculosis (RR-TB) were diagnosed, of which 78% were MDR-TB. Globally, 3·3% of new tuberculosis cases and 18·0% of previously treated cases are RR-TB or MDR-TB.^1^ Only about one of four MDR-TB cases is detected and the patient notified, and of those notified, only 57% achieve treatment success.^1^ Moreover, global deaths due to MDR-TB remain high at 182 000 per year, making it a leading cause of death related to antimicrobial resistance globally.^2^ MDR-TB is undermining progress towards WHO's End TB Strategy goal of a reduction in tuberculosis deaths of 90% of 2015 rates by 2030.^3^

Long and toxic MDR-TB treatment regimens including injectable aminoglycosides, which can cause ototoxicity, contribute to low rates of treatment success and high mortality.^4^ For those affected, these low rates of treatment success can be due to difficulties maintaining long-term adherence, side effects, and severe socioeconomic impacts.^5^ Health system factors also influence MDR-TB treatment success rates, especially in low-income and middle-income countries, where national tuberculosis programmes are frequently under-resourced.^6^ These factors include inadequate and inconsistent drug supply, non-standardised national MDR-TB treatment guidelines, suboptimal monitoring for clinical response and adverse effects, and limited capacity to provide holistic clinical or socioeconomic support for people with tuberculosis and their household members.^7^ Although shorter, all-oral regimens are now the gold standard MDR-TB treatment regimen recommended by WHO, their availability remains scarce and most people with MDR-TB globally receive long regimens.^1^, ^7^

**Research in context**

**Evidence before this study**

We searched PubMed, Embase, Web of Science, Cochrane database, and Google Scholar for systematic reviews and observational, cohort, and randomised controlled trial studies published in English, French, or Spanish between Jan 1, 1980, and June 1, 2021, that reported treatment outcomes of people with multidrug-resistant tuberculosis (MDR-TB). The following search terms were used: (“tuberculosis, multidrug resistant”[MeSH Terms] OR (“tuberculosis”[All Fields] AND “multidrug resistant”[All Fields]) OR “multidrug-resistant tuberculosis”[All Fields] OR (“multi”[All Fields] AND “drug”[All Fields] AND “resistant”[All Fields] AND “tuberculosis”[All Fields]) OR “multi drug resistant tuberculosis”[All Fields]) AND (“treatment outcome”[MeSH Terms] OR (“treatment”[All Fields] AND “outcome”[All Fields]) OR “treatment outcome”[All Fields] OR (“treatment”[All Fields] AND “outcomes”[All Fields]) OR “treatment outcomes”[All Fields]). In pooled analyses, MDR-TB treatment success rates were about 60%, which is lower than the WHO target (≥75%). Factors associated with a higher likelihood of adverse treatment outcomes included male sex, poverty, tuberculosis-related socioeconomic impact, drug or alcohol misuse, underweight, HIV, advanced disease (including pulmonary cavitations and sputum smear positivity), and pre-extensive or extensive drug resistance patterns. WHO's 2019 MDR-TB guidelines recommend against short treatment regimens in people or geographical areas with resistance to pyrazinamide, ethionamide, or prothionamide. Recent evidence suggests shorter, all-oral MDR-TB regimens can achieve treatment success rates of more than 80%. Interpretation was limited by study design heterogeneity and incomplete recording of social and health factors.

**Added value of this study**

To our knowledge, this is the first national cohort study of people with MDR-TB from Sierra Leone, a low-income country with a high tuberculosis burden, and the largest single-country west African MDR-TB cohort reported to date. MDR-TB treatment success rates in Sierra Leone (73%) are comparable to global rates, programmatic rates in other sub-Saharan African countries, and are approaching WHO targets (≥75%). People treated with short (9–11 month) versus long (18–24 month) regimens had better outcomes, despite both involving injectable aminoglycosides. Factors associated with adverse MDR-TB treatment outcomes included economically productive, working age (45–64 years), severe underweight, untreated HIV, chronic lung disease, previous unsuccessful drug-sensitive tuberculosis retreatment, and a long regimen. Most (70%) people with MDR-TB and untreated HIV died, 57% of whom never received tuberculosis treatment. Aminoglycoside-related complete deafness and prothionamide resistance were also independently associated with an adverse outcome.

**Implications of all the available evidence**

MDR-TB is a leading cause of mortality from antimicrobial resistance. People with MDR-TB who do not recover are more likely to transmit the disease and might also develop pre-extensive or extensive drug-resistant tuberculosis, which are associated with high mortality. Our findings have immediate policy and practice implications in Sierra Leone. These include redesign of care pathways to prioritise scarce resources and provide cost-effective and equitable MDR-TB care, integrated with HIV or non-communicable disease care for cases in which this is possible. The findings reinforce the need for vigilant enquiry and audiometry for aminoglycoside-related ototoxicity and roll-out of all-oral regimens. The use of programmatic data from the National TB Programme and the analytical methods employed could be replicated by other national programmes. Our findings appear to support WHO's recommendation to consider alternatives to the short MDR-TB treatment regimen in people or geographical areas with prothionamide resistance.

Sierra Leone is among the 30 high tuberculosis burden countries globally with an estimated incidence of 295 cases per 100 000 population of drug-sensitive tuberculosis and 8·2 cases per 100 000 population of MDR-TB.^1^ Between 1991 and 2002, Sierra Leone had a protracted civil war, which negatively impacted an already under-resourced health system. Between 2000 and 2014, drug-sensitive tuberculosis treatment success rates increased and mortality reduced.^8^ However, the 2014–16 west Africa Ebola virus disease epidemic negatively affected tuberculosis services^9^ and was estimated to cause 2164 additional tuberculosis deaths.^10^ More recently, COVID-19-related health system distortion has led to reduced tuberculosis case notification rates.^11^

MDR-TB diagnosis in Sierra Leone is reliant on smear microscopy and molecular testing and there is a dearth of functional laboratory space in which to perform mycobacteriological culture and phenotypic drug sensitivity testing. Case detection is low at 21% for first-episode GeneXpert-confirmed drug sensitive tuberculosis and 2% for first-episode MDR-TB.^12^

In 2016, WHO approved the use of second-line drugs to treat patients with MDR-TB using a revised short treatment regimen of 9–11 months, but made a conditional recommendation that alternative regimens be used in people with disease resistant to ethionamide, prothionamide, or pyrazinamide.^13^, ^14^ In April 2017, Sierra Leone initiated MDR-TB treatment with the WHO short regimen. Using programmatic data collected since April 2017, we did the first national cohort study of people with MDR-TB in Sierra Leone with the aim of investigating the social and health factors associated with adverse MDR-TB treatment outcomes.

## Methods

### Study design and participants

We did a retrospective cohort study of people notified with MDR-TB between April, 2017, and September, 2019, and admitted to Lakka Hospital. Lakka Hospital is a government-supported hospital that offers care for people with tuberculosis, HIV, and leprosy, situated in Lakka, Western Area Rural District, on the western outskirts of Freetown, Sierra Leone. Any patient with GeneXpert-confirmed RR-TB in Sierra Leone is presumed to have MDR-TB and referred to Lakka Hospital to be admitted for a period of 4–6 months. A small minority of patients (<5%) with positive GeneXpert tests, but without rifampicin resistance, are also referred due to high clinical suspicion of MDR-TB or subsequent Drug Susceptibility Testing (DST) indicating MDR-TB. Discharge is followed by monthly outpatient visits to the hospital to collect tuberculosis medications and for medical evaluation for a further 5–16 months depending on the treatment regimen received. Follow-up and treatment outcome data were collected for all patients included in the cohort until May, 2021.

Sierra Leone national MDR-TB patient categories, adapted from the WHO Drug-resistant TB Treatment Guidelines Update 2016,^15^ are described as follows. Category 1: unsuccessful drug-sensitive tuberculosis treatment regimen for new patients; people who remain sputum-smear-positive or culture-positive for 5 months or longer during drug-sensitive tuberculosis treatment. Category 2: unsuccessful drug-sensitive tuberculosis retreatment regimen; people who are sputum-smear-positive at 5 months or later during their category 2 retreatment, which includes streptomycin in addition to the first-line drugs. Relapse: people who were previously treated for tuberculosis, declared cured or treatment completed at the end of their most recent course of treatment, and are now diagnosed with a recurrent episode of tuberculosis (either a true relapse or a new episode of tuberculosis caused by reinfection). Treatment after loss to follow-up: people who were previously treated for tuberculosis and whose most recent treatment was interrupted for 2 or more consecutive months. New: no previous history of tuberculosis treatment.

This was a pragmatic, opportunistic cohort study that used routinely collected and deidentified programmatic data. All people with MDR-TB notified to the Sierra Leone National TB Programme and admitted to Lakka Hospital from April 1, 2017, to Sept 1, 2019, were eligible to participate. People who were eligible but had no social or health data available (eg, self-discharged or died before data were collected) or were subsequently found to have been misdiagnosed were excluded from the study.

The study was endorsed by the Sierra Leone National TB Programme and verbal consent to analyse routinely collected, deidentified programmatic data was obtained from all participants during their hospital admission as part of routine care. Ethics approval was obtained from the Ethics Review and Scientific Committee of the Ministry of Health and Sanitation, Government of Sierra Leone.

### Procedures

The treatment regimens for patients and their eligibility criteria, and the medications used in each regimen for people with MDR-TB are summarised in the panel.

Panel **MDR-TB short and long treatment regimens in Sierra Leone during the study period**

**Short regimen (9–11 months)**

*Drugs used daily in 4–6 month intensive phase*

• Kanamycin or capreomycin
• Clofazimine
• Moxifloxacin
• Prothionamide
• Isoniazid (high dose, 600–1500 mg per day)
• Ethambutol
• Pyrazinamide

*Drugs used in 5-month continuation phase*

• Moxifloxacin
• Clofazimine
• Ethambutol
• Pyrazinamide

Eligibility criteria for selecting treatment regimen*

• Proven RR-TB or MDR-TB by microbiological or molecular test
• Category 1: unsuccessful drug-sensitive tuberculosis treatment regimen of new patients
• Category 2: Unsuccessful drug-sensitive tuberculosis retreatment regimen
• No eligibility criteria for long regimen are met

**Long regimen (18–24 months)†**

*Drugs used daily in 8–12 month intensive phase*

• Kanamycin or capreomycin
• Levofloxacin
• Prothionamide
• Cycloserine
• Pyrazinamide
• Isoniazid

*Drugs used in 12-month continuation phase*

• Levofloxacin
• Prothionamide
• Cycloserine

Eligibility criteria for selecting treatment regimen*

• Lack of conversion of sputum culture to negative at month 6 of short MDR-TB treatment regimen
• Those previously exposed to second-line drugs or substitutes for second-line drugs (eg, streptomycin), with confirmed Drug Susceptibility Testing resistance to these drugs
• Patients with pre-extensively drug-resistant tuberculosis (indicating resistance to rifampicin, isoniazid, plus quinolones or injectable agents) or extensively drug resistant tuberculosis (indicating resistance to rifampicin, isoniazid, quinolones, and injectable agents)
• Patients with extrapulmonary MDR-TB

**Individualised regimen**

• Injectables switched to bedaquiline‡
• Drugs substituted to alternatives with favourable side-effect profile within the same WHO A, B, or C group

Eligibility criteria for selecting treatment regimen*

• Patients with adverse drug reactions to injectables
• Patients with adverse reactions to other drugs
• Patients with resistance to injectables and other drugs

Drugs, treatment regimens, and eligibility criteria are replicated from the Sierra Leone National TB Programme Guidelines for the treatment of drug-resistant tuberculosis.^16^ MDR-TB=multidrug-resistant tuberculosis. RR-TB=rifampicin-resistant tuberculosis. *To be eligible for this regimen, at least one of these criteria needs to be met. In rare cases, people previously treated for tuberculosis with negative GeneXpert but positive sputum smear or culture, or compatible tuberculosis symptoms, would be treated for MDR-TB with the short regimen at the clinician's discretion. †Patients who switched from a short to long regimen had to restart their treatment from the beginning of the long regimen intensive phase. ‡Bedaquiline became available for use in Sierra Leone on a case-by-case basis in 2018 with Global Fund and National TB Programme funding.

On admission to Lakka Hospital, programmatic data on age, sex, district of residence, occupation, comorbidities (including HIV, diabetes, chronic renal disease, and chronic lung disease), body-mass index (BMI), and social history (including smoking and incarceration history) were recorded. Further details on study definitions of chronic kidney disease, chronic lung disease, and diabetes; and clinical investigation, management, and adverse effect monitoring can be found in the appendix (p 1). Data collected from participants were anonymised, stored in password-protected Excel files on a secure server of the Sierra Leone National TB Programme, and exported to STATA version 15.

MDR-TB treatment outcomes were recorded according to WHO criteria adapted by Sierra Leone's National TB Programme (appendix p 2). Adverse treatment outcomes were defined as death, treatment failure, loss to follow-up, and no evaluation. Treatment success was defined as cure or treatment completion. Participants who were still on treatment at the time of analysis were excluded from adverse treatment outcome analyses. Dates of death were not routinely recorded.

### Statistical analysis

Baseline social and health data were summarised using numbers and percentages, mean with SDs, and median with IQRs, depending on distribution. Age and BMI were recorded as discrete, continuous variables and subsequently converted to WHO-defined categories for analysis.^17^ Age categories were younger than 15 years, 15–24 years, 25–44 years, 45–64 years, and 65 years and older. BMI categories were less than 16.5 kg/m^2^ (severely underweight), 16·5–18·49 kg/m^2^ (underweight), 18·5–24·99 kg/m^2^ (normal weight), and 25 kg/m^2^ and higher (overweight).

Details concerning identification of relevant baseline social and health variables for the regression model can be found in the appendix (p 1, 4–5). Using the “mice” STATA package, we did multiple imputation with chained equations to replace missing values for all potential predictor variables with missing values (<10%).^18^ A univariable analysis was done to estimate the association between each of these variables and adverse treatment outcomes. Unadjusted odds ratios (OR), 95% CIs, and p values were calculated. Subsequently, a multivariable logistic regression including all variables (age, sex, employment, tobacco smoking, BMI, comorbidities [HIV, diabetes, chronic kidney disease, and chronic lung disease], tuberculosis disease severity [chest radiograph findings, sputum smear result], aminoglycoside-related ototoxicity, patient treatment category, and treatment regimen), regardless of their association in univariable logistic regression, was constructed. Adjusted ORs (aOR), 95% CIs, and p values were calculated. Independent variables or subcategories that were associated with adverse treatment outcomes in this multivariable model at a level of p<0·15 were included in a focused model, which was also adjusted for age, sex, and treatment regimen received (eg, short or long). Interaction terms were used to evaluate the effect modification of variables entering the focused multiple logistic regression model with a biologically plausible influence on each other (eg, age and comorbidities), with adverse tuberculosis treatment outcomes using the STATA “mfpigen” command and the likelihood ratio test. p values of less than 0·05 (5%) were considered indicative of a significant interaction.

Missing DST data were above the prespecified threshold of 10% and not included in the imputed multivariable logistic regression model. To investigate key policy issues related to MDR-TB treatment regimens according to DST profile, a sensitivity analysis was done, which consisted of an unimputed univariable and multivariable logistic regression model, which included DST resistance profiles as an independent variable. Given the importance of understanding the influence of HIV co-infection on MDR-TB treatment outcomes, an additional descriptive analysis explored HIV profile, treatment regimen received, and MDR-TB treatment outcomes. Finally, a descriptive analysis compared the social and health characteristics of participants who received the short regimen, long regimen, and no treatment.

This study was reported according to STROBE guidelines. Data were analysed with STATA (version 15).

### Role of the funding source

The funders of the study had no role in study design, data collection, data analysis, data interpretation, or writing of the report.

## Results

Of the 370 people diagnosed with MDR-TB during the study period from April 1, 2017, to Sept 1, 2019, 365 (99%) were eligible for the study (one person was excluded due to a misdiagnosis and four were excluded due to lack of social or health factor data). Of these 365 eligible people, 341 (93%) received treatment (24 received the long MDR-TB treatment regimen and 317 received the short regimen; figure). Participants’ individual social and health characteristics, district poverty level, and tuberculosis notification rates, tuberculosis investigations, categories, treatment regimens, aminoglycoside-related ototoxicity, DST profiles, and proportional adverse outcome rates are summarised in table 1. Participants’ median age was 35 years (IQR 26–45), 263 (72%) of 365 were male and 102 (28%) were female, 240 (66%) were underweight, and 71 (19%) had HIV co-infection of whom 51 (72%) were receiving antiretroviral therapy. Baseline DST was not available for 50 (14%) of 365 participants, which precluded DST imputation. Of the 315 participants who underwent DST at baseline, 303 (96%) were resistant to rifampicin and 258 (82%) were resistant to isoniazid. Additionally, 73 (23%) of 314 were resistant to pyrazinamide, and 56 (18%) of 314 were resistant to ethambutol. Resistance was also seen to prothionamide (35 [11%] of 312), capreomycin (9 [3%] of 313), streptomycin (five [2%] of 313), or quinolones (three [1%] of 314).

**Figure:**
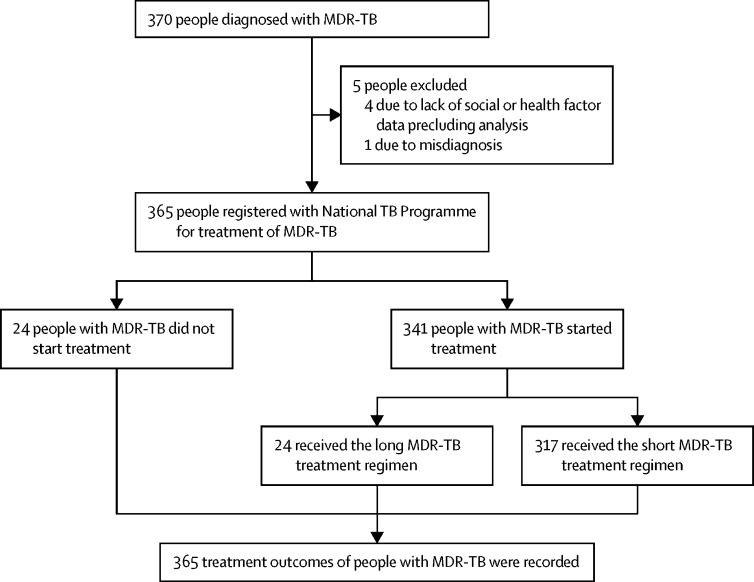
Study profile

**Table 1:** Social and health characteristics of the study population and prevalence of adverse outcomes

		**Patients with MDR-TB (n=365)**	Patients with adverse outcome (n=95)*
**Social characteristics**			
Age, years (median, IQR)		35 (26–45)	37 (28–49)
	0–14	7 (2%)	2/7 (29%)
	15–24	65 (18%)	13/65 (20%)
	25–44	194 (53%)	46/194 (24%)
	45–65	93 (25%)	32/93 (34%)
	65 and older	6 (2%)	2/6 (33%)
Sex			
	Male	263 (72%)	63/263 (24%)
	Female	102 (28%)	32/102 (31%)
Unemployed		104 (28%)	33/104 (32%)
Ever been incarcerated		13/352 (4%)	0
Current or ex-smoker		141 (39%)	33/141 (23%)
Body-mass index, kg/m^2^ (median, IQR)		17 (16–19)	16 (14–18)
	<16·50 (severely underweight)	127/358 (35%)	47/127 (37%)
	16·50–18·49 (underweight)	113/358 (32%)	21/113 (19%)
	18·50–24·99 (normal weight)	112/358 (31%)	18/112 (16%)
	25 or more (overweight)†	6/358 (2%)	3/6 (50%)
Regional diagnostic hub			
	Southern (Bo Regional Hospital)	28 (8%)	9/28 (32%)
	Eastern (Kenema Government Hospital)	30 (8%)	7/30 (23%)
	Western and National (Lakka)	195 (53%)	49/195 (25%)
	North (Makeni Government Hospital)	112 (31%)	30/112 (27%)
District poverty level			
	Tertile 1 (poorest)	195 (53%)	49/195 (25%)
	Tertile 2	107 (29%)	26/107 (24%)
	Tertile 3 (least poor)	63 (17%)	20/63 (32%)
District tuberculosis notification rate (100 000 cases per year)			
	<200	44 (12%)	13/44 (30%)
	200–400	158 (43%)	38/158 (24%)
	>400	163 (45%)	44/163 (27%)
**Health characteristics**			
HIV			
	HIV negative	294 (81%)	67/294 (23%)
	HIV positive on antiretroviral therapy	51 (14%)	13/51 (25%)
	HIV positive not on antiretroviral therapy	20 (5%)	15/20 (75%)
Diabetes		4 (1%)	1/4 (25%)
Chronic kidney disease		12 (3%)	6/12 (50%)
Chronic lung disease		82/346 (24%)	28/82 (34%)
Chest radiograph (n=347)			
	Normal	11/347 (3%)	2/11 (18%)
	Severe tuberculosis on chest radiograph	100/347 (29%)	25/100 (25%)
	Consolidation	108/347 (31%)	30/108 (28%)
	Cavitations	97/347 (28%)	23/97 (24%)
	Effusion	45/347 (13%)	14/45 (31%)
	Nodular	45/347 (13%)	6/45 (13%)
	Fibrosis	44/347 (12%)	13/44 (30%)
	Lymphadenopathy	38/347 (11%)	11/38 (29%)
	Bronchiectasis	22/347 (6%)	9/22 (41%)
	Chronic obstructive pulmonary disease	16/347 (5%)	6/16 (38%)
	Pneumothorax	16/347 (5%)	5/16 (31%)
	Miliary	8/347 (2%)	3/8 (38%)
Sputum smear			
	No smear result	12 (3%)	11/12 (92%)
	Negative	53 (15%)	13/53 (25%)
	+/−	45 (12%)	17/45 (38%)
	1+	61 (17%)	14/61 (23%)
	2+	101 (28%)	17/101 (17%)
	3+	93 (25%)	23/93 (25%)
GeneXpert			
	No GeneXpert result	11 (3%)	11/11 (100%)
	Negative	1 (<1%)	1/1 (100%)
	Positive and rifampicin resistant	353 (97%)	83/353 (24%)
Culture			
	No culture result	38 (10%)	28/38 (74%)
	Negative	64 (18%)	10/64 (16%)
	Positive	263 (72%)	57/263 (22%)
Aminoglycoside-related ototoxicity (n=351)			
	None	326/351 (93%)	72/326 (22%)
	Partial deafness or tinnitus	15/351 (4%)	4/15 (27%)
	Complete deafness	10/351 (3%)	6/10 (60%)
DST			
	No DST result (n=50)	50/50 (100%)	31/50 (62%)
	Rifampicin resistant (n=315)	303/315 (96%)	60/303 (20%)
	Isoniazid resistant (n=315)	258/315 (82%)	54/258 (21%)
	Pyrazinamide resistant (n=314)	73/314 (23%)	19/73 (26%)
	Ethambutol resistant (n=314)	56/314 (18%)	13/56 (23%)
	Prothionamide resistant (n=312)	35/312 (11%)	14/35 (40%)
	Para-aminosalicylic acid resistant (n=314)	10/314 (3%)	1/10 (10%)
	Capreomycin resistant (n=313)	9/313 (3%)	3/9 (33%)
	Streptomycin resistant (n=313)	5/313 (2%)	2/5 (40%)
	Quinolone resistant‡ (n=314)	3/314 (1%)	2/3 (67%)
Treatment category			
	Category 1: unsuccessful first treatment	162 (44%)	40/162 (25%)
	Category 2: unsuccessful retreatment	66 (18%)	21/66 (32%)
	Retreatment after interruption or lost to follow-up	58 (16%)	14/58 (24%)
	Relapse	49 (13%)	14/49 (29%)
	New	30 (8%)	6/30 (20%)
Treatment regimen			
	None	24 (7%)	24/24 (100%)
	Short	317 (87%)	60/317 (19%)
	Long	24 (7%)	11/24 (46%)

Data are n (%), or n/N (%), unless otherwise specified. For cases in which variable data were incomplete, the category n value in parenthesis indicates the number of participants with complete data. Tuberculosis case notifications and poverty levels are those in the district in which the patient was diagnosed before being referred to Lakka Hospital and are taken from nationally published data. Of the 24 patients who received the long regimen, 4 (17%) were started on a short regimen but transferred to a long regimen during the same tuberculosis episode. For chest radiograph findings, multiple findings were possible in the same patient. Sputum smear, GeneXpert, culture, and DST were all recorded at baseline before MDR-TB treatment initiation. MDR-TB=multidrug-resistant tuberculosis. DST=Drug Susceptibility Testing.

* 362 of 365 people with MDR-TB had a recorded outcome because three people within the population cohort were still on treatment at the time of final analysis.

† One participant in the cohort had a body-mass index >30 (obese) and was included in the body-mass index ≥25 (overweight) category.

‡ These three participants with MDR-TB plus quinolone resistance were considered to have pre-extensively drug-resistant tuberculosis; there were no participants in this cohort with pre-extensively drug-resistant tuberculosis as defined by the 2016 WHO definition (ie, MDR-TB plus resistance to any fluoroquinolone and at least one of amikacin, kanamycin, or capreomycin).

Unsuccessful first-line treatment (category 1) was the most common patient category (162 [44%] of 365) followed by unsuccessful retreatment (category 2, 66 [18%] of 365). Most participants (317 [87%]) received a short regimen, 24 (6%) received a long regimen, and 24 (7%) received no treatment. Of the 24 patients who received the long regimen, four (17%) were started on a short regimen but transferred to a long regimen during the same tuberculosis episode. For the purposes of analysis, these patients were grouped with those who received long treatment regimen only.

Outcome analysis by patient category and treatment regimen is shown in table 1 and the appendix (pp 4–6). Overall, 267 (73%) of 365 participants had treatment success, 95 (26%) had an adverse outcome, and three (1%) were not evaluated because they remained on treatment in May, 2021. Of the 95 with adverse outcomes, 75 (79%) died, 12 (13%) were lost to follow-up, and eight (8%) did not have sputum conversion. Higher treatment success rates were seen in new (24 [80%] of 30) and category 1 (121 [75%] of 162) participants and those who received short treatment regimens (254 [80%] of 317) versus long (13 [54%] of 24) regimens. Of those who never started treatment, 21 (88%) of 24 died. Descriptive analysis of monthly sputum smear and culture testing during treatment showed that a higher proportion of participants with adverse MDR-TB treatment outcomes had ongoing smear or culture positivity after month 3 of treatment, than did people with MDR-TB who had treatment success. Proportions of smear positivity, smear negativity, and no sputum sample submitted by month of MDR-TB treatment are shown in the appendix (pp 9–12).

The primary imputed, focused multivariable logistic regression analysis showed that the baseline variables independently associated with higher likelihood of adverse treatment outcomes were age 45–64 years (aOR 2·4, 95% CI 1·2–5·0), severe underweight (aOR 4·2, 1·9–9·3), HIV positive and not on antiretroviral therapy (aOR 10, 2·6–40), chronic lung disease (aOR 2·0, 1·0–4·2), unsuccessful retreatment cases (category 2, aOR 4·3, 1·0–19), and long treatment regimen (aOR 6·5, 2·3–18·0; table 2). People with HIV on antiretroviral therapy had a similar likelihood of adverse treatment outcomes as did people who were HIV negative (aOR 0·94, 0·37–2·4). Participants who had aminoglycoside-related complete deafness (aOR 4·3, 0·86–21·0) or who had chronic kidney disease (aOR 3·5, 0·90–14·0) showed a non-significant association with increased likelihood of adverse treatment outcomes. No independent variables were found to have effect-modifying interactions.

**Table 2:** Multiple imputation univariable and multivariable logistic regression of factors associated with adverse tuberculosis treatment outcome

		**Univariable logistic regression (n=362)**		**Full multivariable logistic regression (n=335)**		**Focused multivariable logistic regression (n=338)**	
		OR (95% CI)	p value	aOR (95% CI)	p value	aOR (95% CI)	p value
**Social characteristics**							
Age, years							
	0–14	1·3 (0·2–6·8)	0·77	0·3 (0·0–3·8)	0·34	0·3 (0·0–3·5)	0·35
	15–24	0·8 (0·4–1·6)	0·53	0·9 (0·4–2·2)	0·77	0·8 (0·3–2·0)	0·64
	25–44	1 (ref)	..	1 (ref)	..	1 (ref)	..
	45–64	1·7 (1·0–3·0)	0·047	2·4 (1·1–5·1)	0·021	2·4 (1·2–5·0)	0·016
	65 and older	1·6 (0·8–9·0)	0·60	1·5 (0·2–13·0)	0·71	1·3 (0·15–11·0)	0·83
Male sex		0·7 (0·4–1·2)	0·17	0·4 (0·2–1·0)	0·044	0·5 (0·3–1·1)	0·081
Unemployed or lowest individual income		1·5 (0·9–2·4)	0·13	1·7 (0·8–3·6)	0·14	1·7 (0·8–3·5)	0·15
Current or ex-smoker		1·0 (0·6–1·7)	0·88	1·4 (0·7–3·0)	0·38	..	..
Nutritional status (body-mass index, kg/m^2^)							
	<16·50 (severely underweight)	2·9 (1·6–5·3)	0·0010	4·3 (1·9–9·7)	0·0010	4·2 (1·9–9·3)	<0·001
	16·50–18·49 (underweight)	1·2 (0·6–2·3)	0·68	1·3 (0·5–3·2)	0·62	1·4 (0·6–3·5)	0·47
	18·50–24·99 (normal weight)	1 (ref)	..	1 (ref)	..	1 (ref)	..
	25 or more (overweight or obese)	4·6 (0·9–25·0)	0·073	3·2 (0·1–73·0)	0·47	3·4 (0·3–47)	0·35
**Health characteristics**							
HIV							
	HIV negative	1 (ref)	..	1 (ref)	..	1 (ref)	..
	HIV positive on antiretroviral therapy	1·1 (0·6–2·3)	0·70	0·9 (0·4–2·4)	0·99	0·9 (0·4–2·4)	0·89
	HIV positive not on antiretroviral therapy	10·0 (3·5–29·0)	<0·001	7·9 (1·8–34·0)	0·0060	10·0 (2·6–40·0)	0·0010
Diabetic		0·9 (1·0–9·1)	0·96	1·2 (0·1–28·0)	0·92	..	..
Chronic kidney disease		2·9 (0·9–9·3)	0·068	3·4 (0·9–14·0)	0·078	3·5 (0·9–14·0)	0·071
Chronic lung disease		2·1 (1·2–3·4)	0·010	2·0 (0·9–4·1)	0·076	2·0 (1·0–4·2)	0·050
**Disease severity**							
Severe chest radiograph		1·2 (0·7–2·1)	0·48	1·0 (0·5–2·2)	0·93	..	..
Baseline smear result							
	Smear negative	1 (ref)	..	1 (ref)	..	..	..
	Smear +/−	1·8 (0·8–4·5)	0·15	1·3 (0·4–4·4)	0·62	..	..
	Smear +	0·9 (0·4–2·1)	0·80	0·8 (0·3–2·7)	0·75	..	..
	Smear ++	0·6 (0·3–1·4)	0·24	0·9 (0·3–2·5)	0·79	..	..
	Smear +++	1·0 (0·5–2·2)	0·97	1·0 (0·4–2·8)	0·99	..	..
Aminoglycoside-related ototoxicity							
	None	1 (ref)	..	1 (ref)	..	1 (ref)	..
	Partial deafness or tinnitus	1·4 (0·4–4·4)	0·59	1·1 (0·3–4·5)	0·93	1·0 (0·2–4·1)	0·99
	Complete deafness	5·1 (1·4–18·0)	0·013	4·1 (0·8–21·0)	0·090	4·3 (0·9–21)	0·076
**Patient treatment**							
Patient treatment category							
	New	1 (ref)	..	1 (ref)	..	1 (ref)	..
	Retreatment after interruption or lost to follow-up	1·3 (0·5–3·9)	0·60	2·4 (0·5–11·0)	0·25	2·4 (0·5–11·0)	0·26
	Relapse	1·6 (0·5–4·8)	0·40	3·4 (0·8–15·0)	0·10	3·5 (0·8–15·0)	0·096
	Category 1: unsuccessful first treatment	1·3 (0·5–3·5)	0·57	1·9 (0·5–7·4)	0·35	1·9 (0·5–7·3)	0·35
	Category 2: unsuccessful retreatment	1·9 (0·7–5·2)	0·24	4·2 (1·0–18·0)	0·059	4·3 (1·0–19·0)	0·049
Treatment regimen							
	Short	1 (ref)	..	1 (ref)		1 (ref)	
	Long	3·6 (1·5–8·4)	0·0030	6·2 (2·2–17·0)	0·0010	6·5 (2·3–18·0)	<0·001
	None	..	..	..	..	..	..

Multiple imputation was used to impute data for variables with incomplete data including current or ex-smoker (n=12 imputed values), chronic lung disease (n=19), body-mass index (n=7), aminoglycoside-related ototoxicity (n=14), and severe chest radiograph (n=18). Participants still on treatment at the time of analysis (3/365, <1%) were recorded as having a treatment outcome of “not evaluated” and excluded from this analysis. The univariable logistic regression model was unadjusted. Full multivariable logistic regression included all independent variables in the model. Variables that were associated with adverse treatment outcome at a level of p≤0·15 were entered into the focused multivariable logistic regression model. 1 (ref) refers to the reference group for categorical variables. All 24 participants who never started treatment perfectly predicted adverse outcome and were not included in the imputed model's odds ratio calculations, leaving an available imputed sample of n=338 participants' data. Incarceration was also not included in the model because 0/13 participants who were currently or previously incarcerated had an adverse outcome. p≤0·1 indicated significance. OR=odds ratio. aOR=adjusted odds ratio.

The unimputed multivariable logistic regression sensitivity analysis broadly replicated the associations described in the main regression model, but also showed that prothionamide resistance (aOR 3·9, 1·5–10·0) and aminoglycoside-related complete deafness (aOR 6·6, 1·3–35·0) were independently associated with adverse MDR-TB treatment outcomes (table 3). In this sensitivity analysis, being HIV positive and not on antiretroviral therapy (aOR 5·8, 95% CI 0·77–44·0) or having chronic lung disease (aOR 2·1, 0·92–4·7) showed a non-significant association with adverse MDR-TB treatment outcomes. Additional descriptive analysis showed that, of people with HIV not on ART, 14 (70%) of 20 died, and eight (57%) of the 14 did not receive any MDR-TB treatment (appendix p 3). Complete data of social and health variables by treatment regimen (no treatment, short regimen, and long regimen) are shown in the appendix (pp 4–5).

**Table 3:** Unimputed univariable and multivariable logistic regression of factors associated with adverse tuberculosis treatment outcome in participants with a DST result available

		**Univariable logistic regression (n=362)**		**Full multivariable logistic regression (n=292)**		**Focused multivariable logistic regression (n=297)**	
		OR (95% CI)	p value	aOR (95% CI)	p value	aOR (95% CI)	p value
**Social characteristics**							
Age, years							
	0–14	1·3 (0·2–6·8)	0·77	3·0 (0·2–46·0)	0·43	2·9 (0·2–39·0)	0·41
	15–24	0·8 (0·4–1·6)	0·53	0·9 (0·3–2·8)	0·87	0·8 (0·3–2·2)	0·65
	25–44	1 (ref)	..	1 (ref)	..	1 (ref)	..
	45–64	1·7 (1·0–3·0)	0·047	3·2 (1·3–8·0)	0·015	2·8 (1·2–6·5)	0·014
	65 years and older	1·6 (0·8–9·0)	0·60	1·3 (0·1–18·0)	0·86	1·0 (0·1–14·0)	0·97
Male sex		0·7 (0·4–1·2)	0·17	0·5 (0·2–1·2)	0·11	0·6 (0·3–1·4)	0·24
Unemployed or lowest individual income		1·5 (0·9–2·4)	0·13	1·0 (0·4–2·6)	0·95	..	..
Current or ex-smoker (n=350)		1·0 (0·6–1·6)	0·96	1·3 (0·5–3·0)	0·50	..	..
Nutritional status (body-mass index, kg/m^2^) (n=355)							
	<16·5 (severely underweight)	3·0 (1·6–5·7)	<0·001	6·0 (2·2–16·0)	<0·001	6·0 (2·3–15)	<0·001
	16·5–18·49 (underweight)	1·2 (0·6–2·3)	0·66	1·4 (0·5–4·5)	0·52	1·7 (0·6–4·9)	0·33
	18·5–24·99 (normal weight)	1 (ref)	..	1 (ref)	..	1 (ref)	..
	25 or more (overweight or obese)	5·1 (1·0–27·0)	0·057	8·2 (0·2–303·0)	0·26	6·7 (0·4–102·0)	0·17
**Health characteristics**							
HIV							
	HIV negative	1 (ref)	..	1 (ref)	..	1 (ref)	..
	HIV positive on antiretroviral therapy	1·1 (0·6–2·3)	0·70	1·1 (0·3–3·4)	0·91	1·2 (0·4–3·7)	0·71
	HIV positive not on antiretroviral therapy	10·0 (3·5–29·0)	<0·001	7·0 (0·8–62·0)	0·081	5·8 (0·8–44·0)	0·089
Diabetic		0·9 (1·0–9·1)	0·96	1·1 (0·0–39·0)	0·96	..	..
Chronic kidney disease		2·9 (0·9–9·3)	0·068	3·0 (0·6–16·0)	0·20	..	..
Chronic lung disease (n=343)		2·2 (1·3–3·8)	0·0050	2·4 (1·0–5·8)	0·063	2·1 (0·9–4·7)	0·078
Disease severity							
Severe chest radiograph (n=349)		1·2 (0·7–2·2)	0·43	0·9 (0·4–2·1)	0·80	..	..
Baseline smear result (n=353)							
	Smear negative	1 (ref)	..	1 (ref)	..	..	..
	Smear +/−	1·9 (0·8–4·5)	0·15	1·1 (0·2–5·6)	0·86	..	..
	Smear +	0·9 (0·4–2·1)	0·80	0·9 (0·2–4·1)	0·88	..	..
	Smear ++	0·6 (0·3–1·4)	0·24	0·9 (0·2–3·6)	0·83	..	..
	Smear +++	1·0 (0·5–2·2)	0·97	1·4 (0·4–5·1)	0·65	..	..
Aminoglycoside-related ototoxicity (n=348)							
	None	1 (ref)	..	1 (ref)	..	1 (ref)	..
	Partial deafness or tinnitus	1·3 (0·4–4·1)	0·69	1·7 (0·4–7·7)	0·49	1·8 (0·4–7·8)	0·43
	Complete deafness	5·2 (1·4–19·0)	0·012	5·5 (1·0–30·0)	0·053	6·6 (1·3–35·0)	0·026
DST							
	Performed	1 (ref)	..	..	..	..	..
	Not performed	6·3 (3·4–12·0)	<0·001	..	..	..	..
DST result*							
Prothionamide resistance (n=312)							
	No	1 (ref)	..	1 (ref)	..	1 (ref)	..
	Yes	3·1 (1·5–6·5)	0·0030	4·1 (1·4–12·0)	0·0090	3·9 (1·5–10·0)	0·0050
Pyrazinamide resistance (n=314)							
	No	1 (ref)	..	1 (ref)	..	..	..
	Yes	1·5 (0·8–2·8)	0·19	1·6 (0·6–4·3)	0·34	..	..
Ethambutol resistance (n=314)							
	No	1 (ref)	..	1 (ref)	..	..	..
	Yes	1·2 (0·6–2·4)	0·59	1·2 (0·4–3·5)	0·71	..	..
Capreomycin resistance (n=313)							
	No	1 (ref)	..	1 (ref)	..	..	..
	Yes	1·9 (0·5–8·0)	0·36	3·9 (0·5–32·0)	0·21	..	..
Streptomycin resistance (n=313)							
	No	1 (ref)	..	1 (ref)	..	..	..
	Yes	2·6 (0·4–16·0)	0·31	7·4 (0·2–283·0)	0·28	..	..
Quinolone resistance (n=314)							
	No	1 (ref)	..	1 (ref)	..	..	..
	Yes	7·6 (0·7–85·0)	0·099	2·8 (0·1–107·0)	0·57	..	..
Para-aminosalicylic acid resistance (n=314)							
	No	1 (ref)	..	1 (ref)	..	..	..
	Yes	0·4 (0·1–3·4)	0·41	0·5 (0·0–5·3)	0·53	..	..
**Patient treatment**							
Patient treatment category							
	New	1 (ref)	..	1 (ref)	..	1 (ref)	..
	Retreatment after interruption or lost to follow-up	1·3 (0·5–3·9)	0·60	3·3 (0·4–27·0)	0·25	3·1 (0·4–22·0)	0·25
	Relapse	1·6 (0·5–4·8)	0·40	5·9 (0·8–44·0)	0·082	4·7 (0·7–32·0)	0·12
	Category 1: unsuccessful first treatment	1·3 (0·5–3·5)	0·57	2·3 (0·3–17·0)	0·39	2·0 (0·3–13·0)	0·47
	Category 2: unsuccessful retreatment	1·9 (0·7–5·2)	0·24	4·6 (0·6–35·0)	0·14	4·7 (0·7–32·0)	0·12
Treatment regimen							
	Short	1 (ref)	..	1 (ref)	..	1 (ref)	..
	Long	3·6 (1·5–8·4)	0·0030	5·1 (1·4–19·0)	0·013	6·2 (1·9–21·0)	0·0030
	None	..	..	..	..	..	..

For cases in which variable data were incomplete, the n value in parenthesis indicates the number of participants with complete data for that particular variable. Participants still on treatment at the time of analysis (3/365, <1%) were recorded as having a treatment outcome of “not evaluated” and were excluded from this analysis. The univariable logistic regression model was unadjusted. Full multivariable logistic regression included all independent variables in the model. Variables that were associated with adverse treatment outcome at a level of p≤0·1 were entered into the focused multiple logistic regression model. 1 (ref) refers to the reference group for categorical variables. Participants who never started treatment or did not have Drug Susceptibility Testing perfectly predicted adverse outcome and were not included in the model's odds ratio calculations. Incarceration was also not included in the model because 0/13 participants who had been incarcerated had an adverse outcome. p≤0·15 indicated significance. OR=odds ratio. aOR=adjusted odds ratio. DST=Drug Susceptibility Testing.

* Resistance in addition to rifampicin and isoniazid.

## Discussion

To our knowledge, this is the first study describing the characteristics and treatment outcomes of the national MDR-TB cohort in Sierra Leone since WHO short treatment regimens were rolled out in April, 2017, and the largest single-country west African cohort of people with MDR-TB reported to date. Treatment success rates in this cohort of people with MDR-TB in Sierra Leone were similar to those seen in programmatic settings such as Tanzania^19^ and Uganda.^20^ However, they were lower than those seen in studies including STREAM.^21^ In our cohort, those receiving the long treatment regimen were more likely to have adverse outcomes than were those receiving the short regimen, which has also been described in an unpublished programmatic report from Sierra Leone.^22^ However, there was significant heterogeneity in treatment outcomes among people with MDR-TB and we demonstrated that several individual-level social and health factors routinely collected at treatment initiation identified people at highest risk of adverse outcomes. The primary imputed analysis showed that age 45–64 years, severe underweight, untreated HIV, previous unsuccessful drug-sensitive tuberculosis retreatment, chronic lung disease, and long regimen were all independently associated with a higher likelihood of adverse MDR-TB treatment outcomes. The unimputed sensitivity analysis showed that prothionamide resistance and aminoglycoside-related complete deafness were also independently associated with adverse outcomes. These data could be used to support the design and implementation of future targeted interventions to support the most clinically vulnerable. Such interventions might include nutritional support, integrated HIV-TB services, linkage with non-communicable disease management, adverse event monitoring, and targeted active case-finding aiming to mitigate adverse MDR-TB outcomes and increase tuberculosis cure rates in Sierra Leone. Our findings also re-emphasise the urgent need for roll-out of safe and effective short, all-oral regimens in low-income and middle-income countries for the treatment of MDR-TB, increased DST capacity and implementation and, while more evidence is collected, support WHO's decision to recommend against short regimens in people with ethionamide or prothionamide resistance.

Worse outcomes with the long regimen might relate to challenges adhering to MDR-TB treatment, including adverse effects such as the aminoglycoside-related ototoxicity seen in our cohort, which was independently associated with adverse outcomes in the sensitivity analysis. Other studies such as meta-analyses suggest difficulties adhering to long DR-TB regimens can be mitigated by direct observation treatment throughout treatment.^23^ Although direct observation treatment is recommended and implemented in Sierra Leone during the initiation phase, patients are not regularly monitored during the ambulatory community-based continuation phase. In 2021, to complement monthly visits to the MDR-TB health-care facility for clinical review and sputum submission, the Sierra Leone National TB Programme implemented a more decentralised approach to care. This approach has involved opening an MDR-TB treatment unit in the northern region; trained nurses following up people with MDR-TB after discharge until treatment completion through regular home visits, which include checking for symptoms of drug-related toxicity; and covering costs for travel to the clinic and sometimes providing food baskets. Additionally, the frequency of kidney, liver, and full blood count blood tests and investigations to monitor for drug toxicity (eg, clinical enquiry and audiometry for aminoglycoside-related ototoxicity) during treatment is increasing. More robust empirical evidence is needed on the differential impact on outcomes of community-based or health facility-based direct observation treatment, treatment supporters, and incentives and socioeconomic support.^24^

A key health factor independently associated with adverse outcomes was untreated HIV. High rates of mortality in HIV-positive people with MDR-TB have been seen in STREAM (17·5%)^25^ and Trebucq and colleagues’ cohort study of people with MDR-TB from nine African countries (19·8%), especially with concomitant underweight.^26^ We did not collect data on CD4 count or time to death but hypothesise that newly diagnosed advanced HIV with disseminated tuberculosis disease was responsible for the high mortality seen in our cohort. Importantly, the effect of HIV on adverse MDR-TB treatment outcomes in our study was attenuated by being on antiretroviral therapy, demonstrating the importance of timely HIV diagnosis, integrated HIV-TB services, and access to appropriate antiretroviral therapy. Furthermore, chronic lung disease was significantly associated with adverse outcomes and chronic kidney disease showed a non-significant association, limited by the small number of patients with a diagnosis (n=12). These findings demonstrate the importance of a holistic approach to the management of tuberculosis with linkage to non-communicable disease management services in appropriate cases.

In our cohort, DST resistance to prothionamide was shown to be independently associated with adverse outcome. WHO's 2019 MDR-TB guidelines recommend against short treatment regimens in people with resistance to pyrazinamide, ethionamide, or prothionamide (or areas with high prevalence of such resistance) due to findings from an individual patient data meta-analysis.^7^, ^14^ By contrast, STREAM and Trebucq and colleagues’ cohort study suggested worse outcomes in people with quinolone and isoniazid resistance. Although our findings appear to support WHO's conditional recommendation, DST of prothionamide might be unreliable; the drug has a role in reducing acquisition of drug resistance, and we cannot account for the mechanism by which prothionamide—unique among all MDR-TB drugs with reported DST profile—could contribute to adverse MDR-TB treatment outcomes. We also showed that people with previous unsuccessful drug-sensitive tuberculosis retreatment were at higher risk of adverse outcomes than were other MDR-TB patient groups, potentially because of more advanced disease following prolonged inappropriate first-line drug treatment.

At treatment initiation, two-thirds of our cohort were underweight and a third were severely underweight; a factor that was found to be independently associated with adverse outcomes. This result is challenging to interpret considering that food insecurity, malnutrition, and underweight can contribute to the likelihood of developing tuberculosis and, conversely, untreated or advanced tuberculosis can compound underweight and be associated with a higher likelihood of adverse outcomes.^27^ Although an important predictor of outcome, examination of weight gain during treatment among our cohort was not possible due to inconsistent measurement following treatment initiation. Another social driver of adverse MDR-TB outcomes in this cohort was being of an economically productive age. In other settings, out-of-pocket costs and lost income have been associated with delay in diagnosis and treatment, advanced disease, and adverse treatment outcomes.^28^ Addressing these issues will probably require active case finding and broader, holistic support. Socioeconomic or nutritional support have been shown to increase tuberculosis treatment success^29^ or proxy measures of tuberculosis treatment success such as sputum conversion.^30^ However, neither was considered in the National Strategic Plan for TB (2016–2020) in Sierra Leone and more evidence is required on which support packages can improve MDR-TB outcomes in this setting.

This study had several limitations. First, we used routinely collected, incomplete programmatic data. Multiple imputation replaced missing data in cases for which this was appropriate, but this was not possible for certain variables, including DST results. However, this limitation points to a wider issue of a paucity of available MDR-TB data. For example, unlike other countries in west Africa, no DR-TB prevalence study has been done in Sierra Leone. Second, certain comorbidities, such as chronic lung disease, were defined pragmatically without formal diagnosis, which might have influenced the estimates of their association with MDR-TB treatment outcomes. Additionally, data on duration of symptoms, time from diagnosis to treatment initiation, alcohol and elicit substance use, full blood count, and renal and liver function tests were not routinely recorded. Such data could be useful in future analyses to determine the effect of treatment delays, risk behaviours, and organ or system dysfunction on treatment outcomes. Third, although poverty is known to be a major social determinant of tuberculosis, health-care access, and treatment outcomes, the minimal socioeconomic data routinely collected included only self-reported employment and district of residence, both of which are inaccurate measures of poverty in this setting. This situation is similar to many other countries both in sub-Saharan Africa and globally and presents a substantial challenge in terms of equity assessment of tuberculosis service provision for people with tuberculosis and MDR-TB by poverty level. Fourth, although there were clinical efforts to monitor and assess patients for side-effects of tuberculosis medications, full side-effect profiles were anecdotally undermeasured and under-reported. Finally, the associations of long treatment regimen and prothionamide with adverse MDR-TB treatment outcome might have been biased by additional unmeasured confounders.

In conclusion, we have presented treatment outcomes of people with MDR-TB in Sierra Leone and showed how adverse outcomes are associated with individual-level social and health data. Our findings can be used to inform the design of MDR-TB treatment services in Sierra Leone and other low-income and middle-income countries, and are even more pertinent given the negative impact of the COVID-19 pandemic and its mitigation measures on access to, quality, and coverage of tuberculosis services. These issues, coupled with the predicted increases in tuberculosis morbidity, mortality, and potentially resistance, will challenge progress towards equitable provision of tuberculosis services in Sierra Leone over the coming decade.

## Data sharing

The deidentified National TB Programme data used in this study, and a relevant data dictionary defining each field in the set, can be obtained by contacting the first author and MDR-TB Program Lead at Ministry of Health and Sanitation Sierra Leone, Rashidatu Fouad Kamara (shidakay700@gmail.com).

## Declaration of interests

TW is supported by grants from the Wellcome Trust, UK (209075/Z/17/Z) and the Medical Research Council, Department for International Development, and Wellcome Trust (Joint Global Health Trials, MR/V004832/1). All other authors declare no competing interests.
