# Supplementary material for: Social and health factors associated with adverse treatment outcomes among people with multidrug-resistant tuberculosis in Sierra Leone: a national, retrospective cohort study
Source: Lancet Glob Health. 2022 Mar 15;10(4):e543–54. doi: 10.1016/S2214-109X(22)00004-3 (PMC8938764; doi:10.1016/S2214-109X(22)00004-3)
Supplement: Supplementary appendix [file mmc1.pdf]

THE LANCET
Global Health

Supplementary appendix

This appendix formed part of the original submission and has been peer reviewed. We post it as supplied by the authors.

Supplement to: Kamara RF, Saunders MJ, Sahr F, et al. Social and health factors associated with adverse treatment outcomes among people with multidrug-resistant tuberculosis in Sierra Leone: a national, retrospective cohort study. *Lancet Glob Health* 2022; **10:** e543–54.

Supplementary online appendix to accompany Kamara *et al* "Social and health factors associated with adverse treatment outcomes among people with multidrug-resistant tuberculosis in Sierra Leone: a national, retrospective cohort study" published in Lancet Global Health, March 2022

Contents

Pages 1-2, Methods: Additional detail on microbiological testing, treatment regimens, and social and health data
Page 3, Supplementary Table 1: HIV profile, treatment regimen, and treatment outcome
Pages 4-6 Supplementary Table 2: Health and social characteristics and adverse outcomes of cohort
Pages 7-8, Supplementary Figures 1a/b: TB treatment outcome of people with MDR-TB by patient category and treatment regimen
Pages 9-12, Supplementary Figures 2a/b and 3a/b: Sputum smear and culture by month of treatment and treatment outcome

Microbiological testing and MDR-TB treatment regimens: Baseline sputum samples collected from people with GeneXpert-confirmed MDR-TB were sent for DST, solid culture, and liquid culture, to a supranational laboratory in Borstel, Germany. Based on these results, patients either continued the short regimen or were switched to the long regimen (Table 1). In cases of patients who reported ototoxicity to the injectable agents (e.g. kanamycin / capreomycin), treatment was individualized through a switch to bedaquiline. Treatment continues at the hospital during the intensive phase of four to six months until the person with MDR-TB has two consecutive negative sputum smears and one negative mycobacterial solid culture. At that time, if clinically stable, they are discharged home for ambulatory care. Lack of sputum culture conversion by month six of treatment is considered unsuccessful treatment and, in patients on the short regimen, requires a switch to restart treatment from the start of the long regimen.

Social and health data: Chronic kidney disease was determined at baseline by self-reporting or medical records. Where possible, monthly renal function and urine dipstick tests were done. Chronic lung disease was pragmatically defined at baseline by self-reporting, medical records, or chest radiograph changes of chronic obstructive pulmonary disease, pulmonary fibrosis, and/or bronchiectasis. Diabetes was determined at baseline by self-reporting, confirmation of use of anti-diabetic medications, medical records, random venous plasma glucose concentration ≥11.1 mmol/l, and/or a fasting plasma glucose concentration ≥7.0 mmol/l. Spirometry, HbA1c and oral glucose tolerance tests were not routinely done. HIV and antiretroviral therapy (ART) use were recorded. HIV testing was offered if HIV status was unknown or previously negative. If a participant had a new positive HIV test, they were referred to an HIV physician and provided with HIV treatment, usually within two to four weeks from diagnosis, according to Sierra Leone National HIV Guidelines. TB and HIV services are integrated at Lakka hospital. Chest radiographs were performed routinely at baseline and, when clinically indicated, repeated. Full blood count and liver function tests were sent at baseline where possible and if clinically indicated thereafter. Sputum mycobacterial culture with drug susceptibility testing (DST) was performed monthly during treatment where possible. People with MDR-TB on bedaquiline had baseline electrocardiograms, which were repeated monthly, or when requested by physicians. A weekly update of the national MDR-TB register was done by the clinical team and the patients' charts were updated during thrice-weekly hospital ward rounds, including any adverse reaction to TB medications. All participants started on treatment had baseline audiometry testing. Participants reporting symptoms consistent with ototoxicity such as hearing impairment were referred for audiometry and, where appropriate, switched to an all-oral aminoglycoside-sparing and bedaquiline-containing regimen.

Identification of social and health variables for the regression model: In line with our related research, we used an adapted version of WHO's medication adherence framework[1,2] to identify relevant baseline social and health variables that had a plausible or proven biological and epidemiological association with adverse treatment outcome and were collected by the NTP and available for analysis. These included age, sex, employment, incarceration, smoking history, BMI, comorbidities (HIV, diabetes, chronic kidney disease), chronic lung disease, severity of disease (defined by cavitating or miliary disease on chest radiograph), baseline smear positivity, and patient category (Box 1).

Supplementary Box 1: Sierra Leone NTP MDR-TB Treatment Outcomes

**Treatment outcomes**	**Definitions**
**Cure**	A patient who has completed treatment and has been had three consecutive negative cultures taken at least thirty days apart
**Treatment completed**	A patient who has completed treatment, but does not meet the definition for cure due to lack of bacteriologic results (i.e. less than three negative cultures taken at least 30 days apart after the intensive phase)
**Treatment success**	The sum of cured and treatment completed
**Death**	A patient who dies from any cause while on MDR-TB treatment
**Loss to follow-up**	A patient who interrupts MDR-TB treatment for two or more consecutive months for any reason
**Treatment failure***	Treatment terminated or need for permanent regimen change of at least two anti-TB drugs because of:<br>- Lack of sputum smear or culture conversion by the end of the intensive phase;<br>- Bacteriological reversion to positive in the continuation phase following prior conversion to negative;<br>- Evidence of additional acquired resistance to fluoroquinolones or second-line injectable drugs<br>- Adverse drug reactions (ADRs)
**No evaluation**	No TB treatment outcome recorded in the NTP treatment register

Legend: Treatment outcomes for patients were broadly aligned with the 2020 WHO revised framework for treatment outcome reporting.[3] **While we acknowledge the term "treatment failure" could potentially be stigmatizing against people with TB and MDR-TB, we use it here to be aligned with the nomenclature in both Sierra Leone NTP and WHO guidance of this specific treatment outcome definition.*

Supplementary Table 1: HIV profile, treatment regimen, and MDR-TB treatment outcome (n=365)

	HIV negative n=294 (%)	HIV positive and on ART at time of TB diagnosis n= 51 (%)	HIV positive and not on ART at time of TB diagnosis n=20 (%)
Treatment regimen			
Short	265 (90)	40 (78)	12 (60)
Long	14 (5)	10 (20)	0 (0)
None	15 (5)	1 (2)	8 (40)
Treatment outcome			
Cured	204 (69)	30 (59)	4 (20)
Treatment completed	20 (7)	8 (16)	1 (5)
Died	50 (17)	11 (22)	14 (70)*
Lost to follow-up	10 (3)	1 (2)	1 (5)
Treatment failure	7 (2)	1 (2)	0 (0)
Not evaluated	3 (1)	0 (0)	0 (0)

*Of people with MDR-TB and untreated HIV who died, 8/14 (57%) received no treatment.

Supplementary Table 2: Health and social characteristics of the study population (n=365) and prevalence of adverse MDR-TB treatment outcome

	Short regimen (%)	Long regimen (%)	No treatment (%)
Overall cohort	317/365 (87)	24/365 (6.5)	24/365 (6.5)
Social characteristics			
Age in years (median, IQR)	35 (26-45)	35 (25-42)	40 (29-50)
Age group 0-14 years	5 (2)	1 (4)	1 (4)
Age group 15-24 years	58 (18)	5 (21)	2 (8)
Age group 25-44 years	169 (53)	14 (58)	11 (46)
Age group 45-65 years	79 (25)	4 (17)	10 (42)
Age group 65 years and older	6 (2)	0 (0)	0 (0)
Male sex	231 (73)	14 (58)	18 (75)
Unemployed	86 (27)	8 (33)	10 (42)
Ever been incarcerated (n=352)	11 (3)	2 (8)	0 (0)
Current or ex-smoker	125 (40)	10 (42)	6 (43)
Nutritional status			
BMI (median, IQR) (n=358)	17 (16-19)	18 (15-19)	17 (13-20)
BMI <16.5 (Severely underweight)	110 (35)	8 (33)	9 (45)
BMI 16.5-18.49 (Underweight)	101 (32)	8 (33)	4 (20)
BMI 18.5-24.99 (Normal weight)	99 (32)	8 (33)	5 (25)
BMI 25 or more (Overweight)**	4 (1)	0 (0)	2 (10)
Regional diagnostic hub			
Southern (Bo Regional Hospital)	26 (8)	0 (0)	2 (8)
Eastern (Kenema Government Hospital)	29 (9)	0 (0)	1 (4)
Western and National (Lakka)	164 (52)	17 (71)	14 (58)
North (Makeni Government Hospital)	98 (31)	7 (29)	7 (29)
District poverty level			
Poorer	164 (52)	17 (71)	14 (58)
Poor	97 (31)	2 (8)	8 (33)
Less Poor	56 (18)	5 (21)	2 (8)
District TB notification rate (100,000/year)			
<200	38 (12)	1 (4)	5 (21)
200-400	142 (45)	7 (29)	9 (38)
>400	137 (43)	16 (67)	10 (42)
Health characteristics			
Comorbidities			
HIV			
HIV negative	265 (84)	14 (58)	15 (63)
HIV positive on ART	40 (13)	10 (42)	1 (4)
HIV positive not on ART	12 (4)	0 (0)	0 (0)
Diabetic	4 (1)	0 (0)	0 (0)
Chronic kidney disease	10 (3)	2 (8)	0 (0)
Chronic lung disease (n=346)	74 (24)	2 (8)	6 (43)
Severe TB on chest radiograph (n=347)	87 (28)	8 (33)	5 (36)
Sputum smear			
No smear result	3 (1)	0 (0)	9 (38)

Negative	45 (14)	4 (17)	4 (17)
+/-	35 (11)	4 (17)	6 (25)
1+	54 (17)	3 (13)	4 (17)
2+	97 (31)	4 (17)	0 (0)
3+	83 (26)	9 (38)	1 (4)
GeneXpert			
No GeneXpert result	3 (1)	0 (0)	8 (33)
Negative	1 (0.5)	0 (0)	0 (0)
Positive and rifampicin resistant	313 (89)	24 (100)	16 (67)
Culture			
No culture result	17 (5)	3 (13)	18 (75)
Negative	56 (18)	5 (21)	3 (13)
Positive	244 (77)	16 (67)	3 (13)
Aminoglycoside-related ototoxicity (n=351)			
None	293 (93)	21 (88)	12 (92)
Partial deafness / tinnitus	14 (4.5)	1 (4)	0 (0)
Complete deafness	7 (2.5)	2 (8)	1 (8)
Direct Susceptibility Testing			
No direct susceptibility test result (n=50)	29 (9)	2 (8)	19 (80)
Rifampicin Resistant (n=315)	276 (96)	22 (100)	5 (2)
Isoniazid Resistant (n=315)	237 (82)	18 (82)	3 (60)
Pyrazinamide Resistant (n=314)	64 (22)	7 (33)	2 (40)
Ethambutol Resistant (n=314)	50 (17)	5 (24)	1 (20)
Prothionamide Resistant (n=312)	28 (10)	7 (33)	0 (0)
PAS Resistant (n=314)	9 (3)	1 (5)	0 (0)
Capreomycin Resistant (n=313)	7 (2.5)	2 (10)	0 (0)
Streptomycin Resistant (n=313)	4 (1)	0 (0)	1 (17)
Quinolone Resistant*** (n=314)	1 (0.5)	1 (5)	1 (14)
Treatment Category			
Unsuccessful first treatment (CAT 1)	134 (42)	16 (67)	12 (50)
Unsuccessful re-treatment (CAT 2)	61 (19)	2 (8)	3 (13)
Retreatment after interruption/LTFU	53 (17)	1 (4)	4 (17)
Relapse	44 (14)	2 (8)	3 (13)
New	25 (8)	3 (13)	2 (8)
Treatment Outcome			
Cured	229 (72)	9 (38)	0 (0)
Treatment completed	25 (8)	4 (17)	0 (0)
Died	48 (15)	6 (25)	21 (88)
Lost to follow-up	8 (3)	1 (4)	3 (13)
No culture conversion	4 (1)	4 (17)	0 (0)
Not evaluated	3 (1)	0 (0)	0 (0)

Legend: Where variable data was incomplete, the category n value in parenthesis indicates the number of participants with complete data. TB case notifications and poverty levels are those in the district in which the patient was diagnosed before being referred to Lakka Hospital and are taken from nationally published data. Of the 24 patients who received long regimen, 4/24 (17%) were started on a short

regimen but transferred to a long regimen during the same TB episode. A cell containing a hyphen indicates not applicable and in the case of chest radiograph findings this relates to multiple findings being possible in the same patient. Abbreviations: IQR = interquartile range; BMI = body mass index; CAT 1 = Category 1; CAT 2 = Category 2; PAS = para-aminosalicylic acid; LTFU = lost to follow up. Sputum smear, Xpert, culture and DST are all those at baseline. *362/365 people with MDR-TB had a recorded outcome because three people within the population cohort were still on treatment at the time of final analysis. **One participant in the cohort had a BMI >30 (obese) and was included in the BMI ≥25 (overweight) category ***These three participants with MDR-TB plus quinolone resistant were considered to have pre-XDR TB, there were no participants in this cohort with XDR-TB as defined by the 2016 WHO definition (i.e. MDR-TB plus resistance to any fluoroquinolone and at least one of amikacin, kanamycin, or capreomycin).

Supplementary Figure 1a: MDR-TB treatment outcome of people with MDR-TB by patient category (n=365)

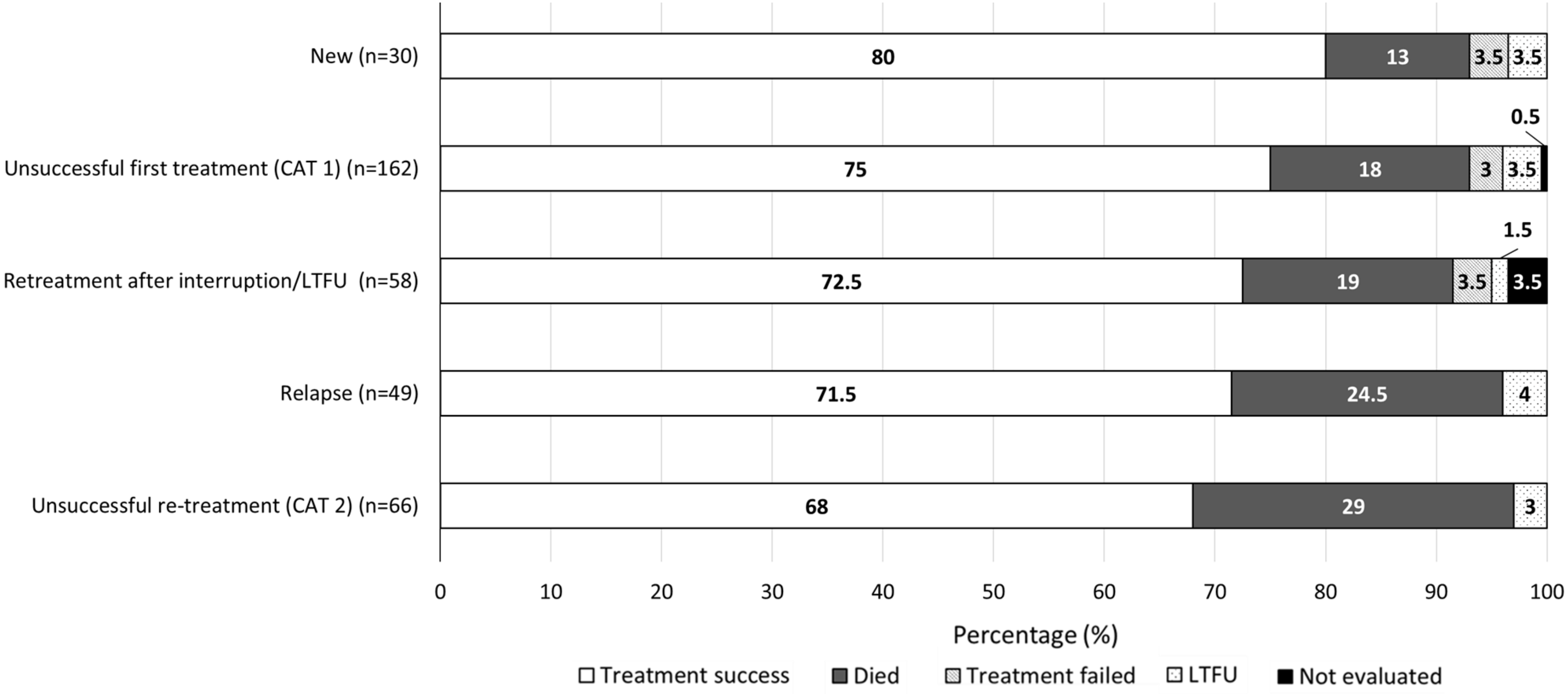

Legend: Treatment outcomes were defined according to World Health Organisation criteria,20 patient categories and treatment regimens were defined by the Sierra Leone National TB Program (see Methods). The figure shows the percentage of people with MDR-TB with key outcomes including treatment success, death, treatment failure, loss-to-follow-up (LTFU), and no evaluation. All participants classified as "not evaluated" (3/365, 0.8%) were still on treatment at the time of analysis.

Supplementary Figure 1b: MDR-TB treatment outcome of people with MDR-TB by treatment regimen (n=365)

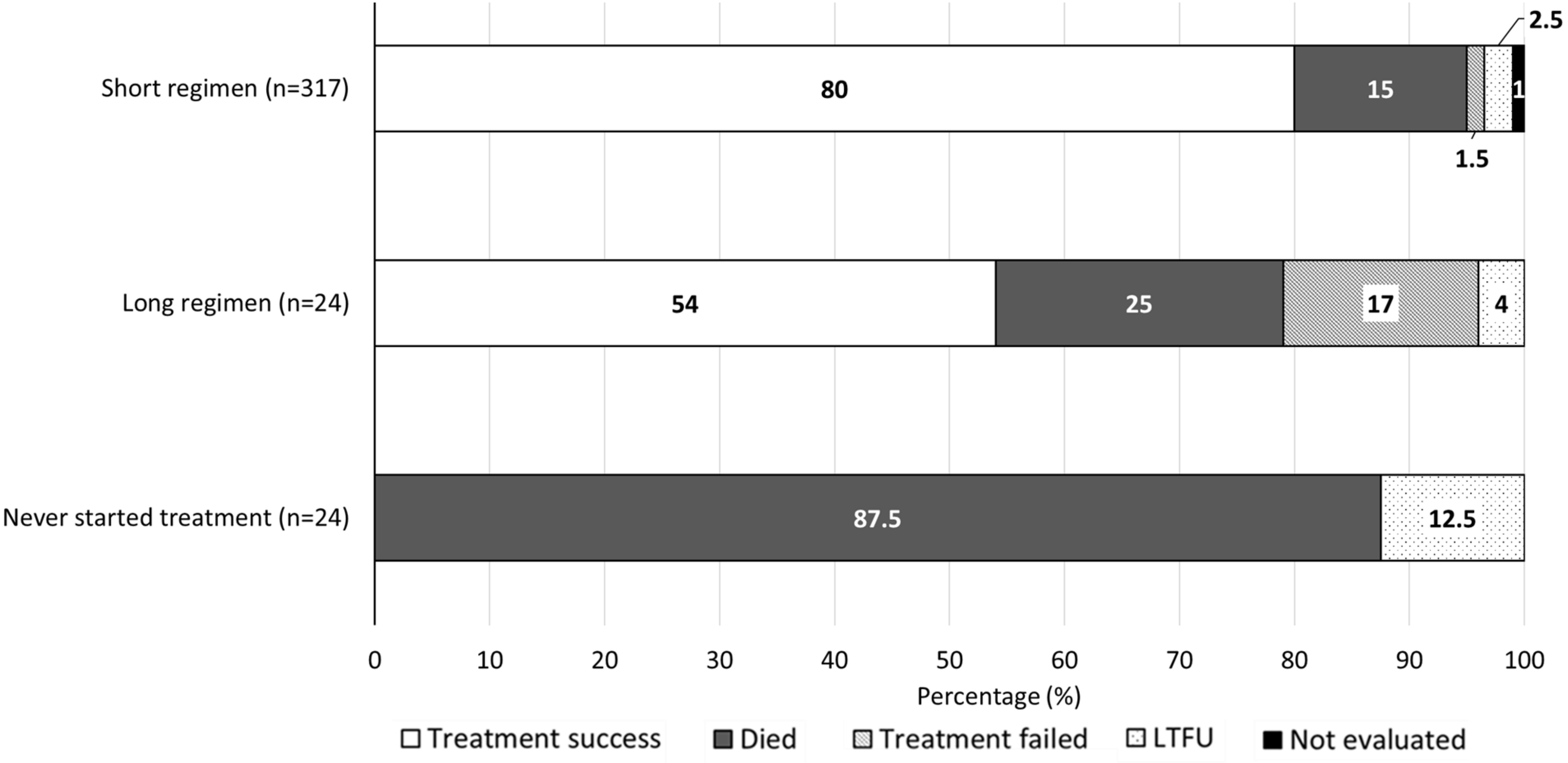

Legend: Treatment outcomes were defined according to World Health Organisation criteria, patient categories and treatment regimens were defined by the Sierra Leone National TB Program (see Methods). The figure shows the percentage of people with MDR-TB with key outcomes including treatment success, death, treatment failure, loss-to-follow-up (LTFU), and no evaluation. Of those on the long regimen, 4/24 (17%) had failed the short regimen and been converted to the long regimen, of whom 2/4 (50%) died and 2/4 (50%) had treatment success. All participants classified as “not evaluated” (3/365, 0.8%) were still on treatment at the time of analysis and received the short regimen.

Supplementary Figure 2a: Ziehl-Nielsen Smear Microscopy results by month of MDR-TB treatment and treatment success vs adverse outcome (n=362)

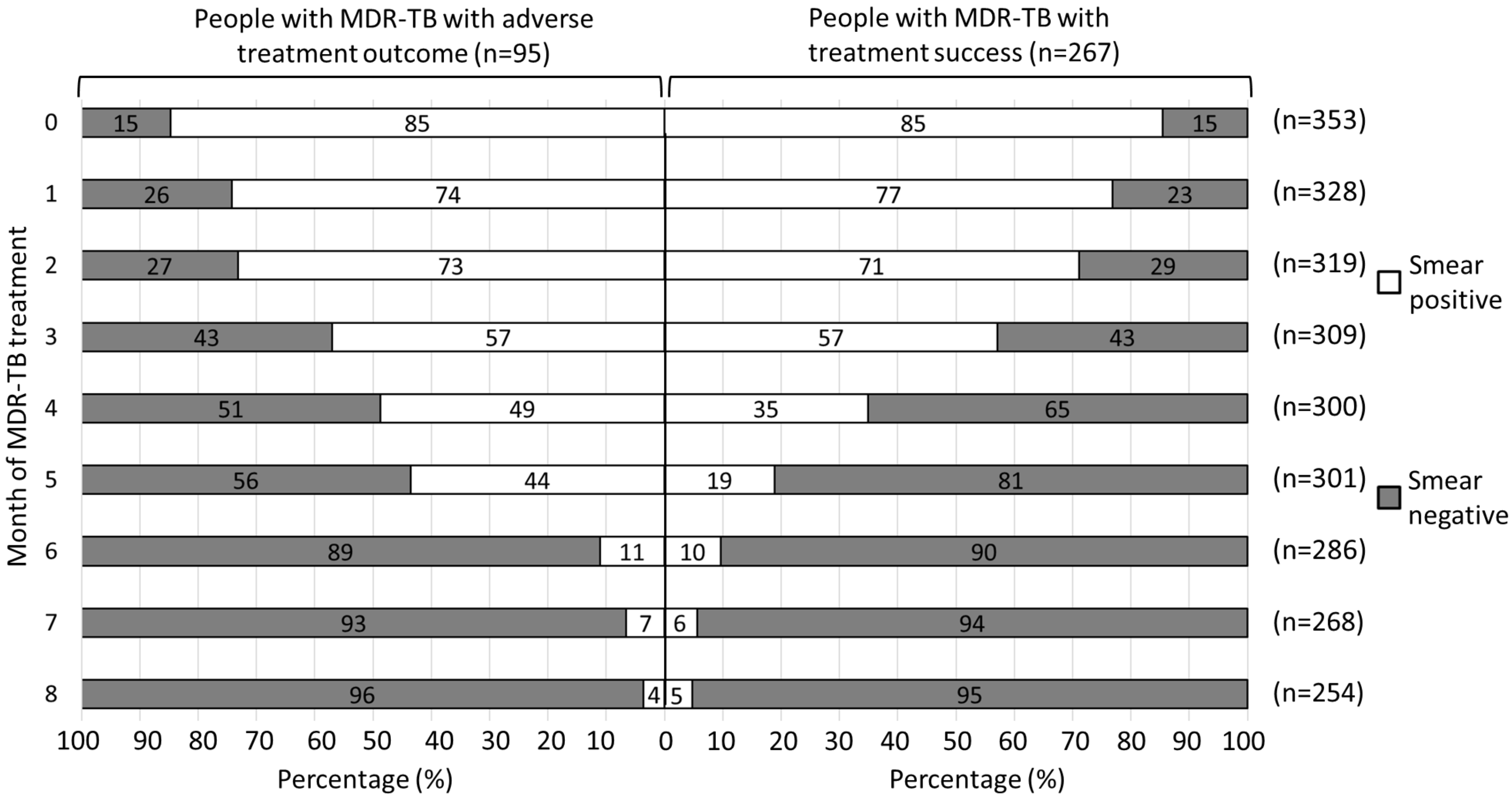

Legend: This figure shows only the monthly smear microscopy results of participants who submitted sputum, the number of which is indicated by the repeated n values in parenthesis on the right side of the figure. Supplementary Figure 1a shows percentages of participants with positive sputum results, negative sputum results, and no sputum result.

Supplementary Figure 2b: Mycobacterial culture result by month of MDR-TB treatment and treatment success vs adverse outcome (n=362)

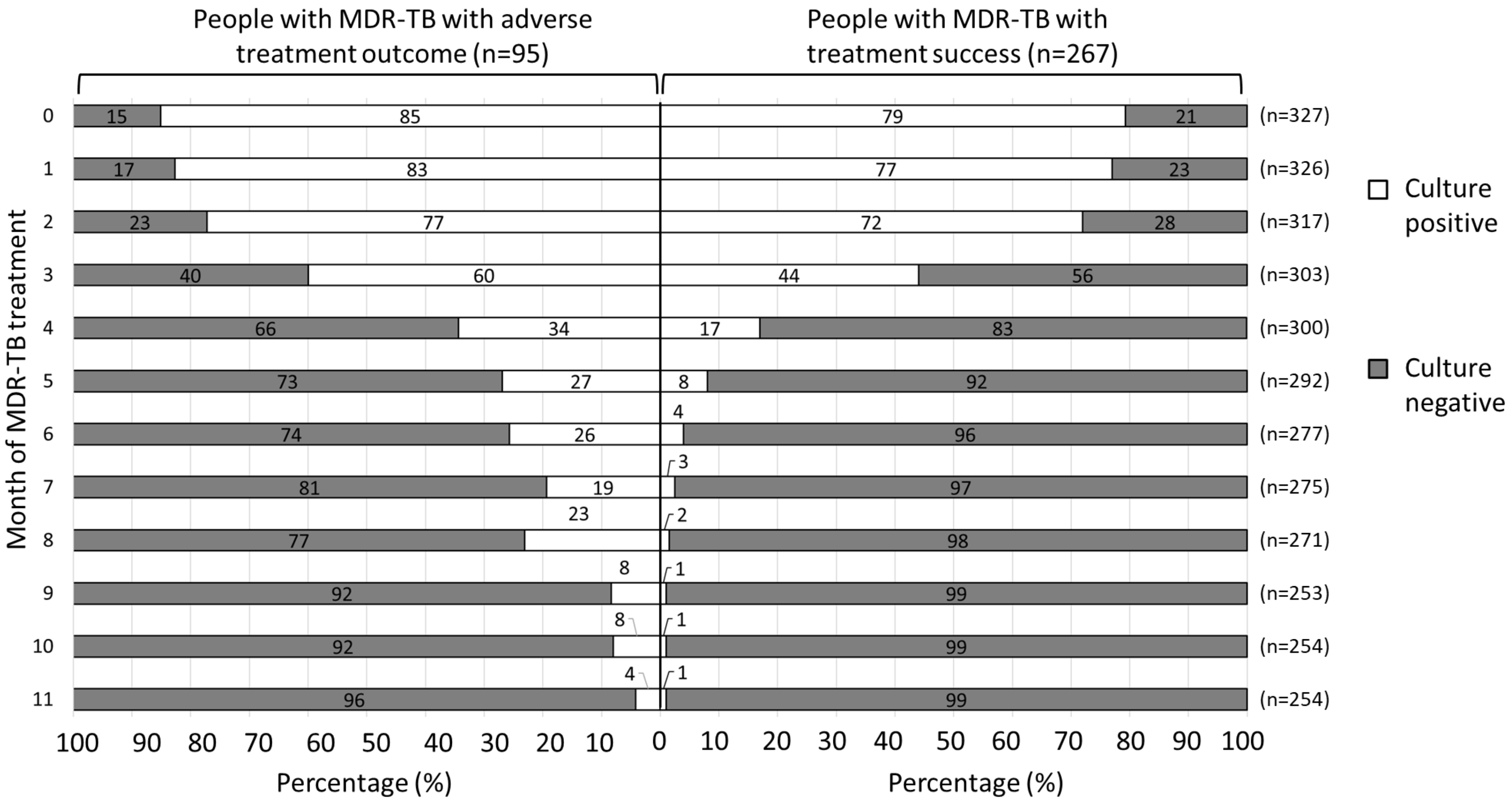

Legend: This figure shows only the monthly mycobacterial culture results of participants who submitted sputum, the number of which is indicated by the repeated n values in parenthesis on the right side of the figure. Supplementary Figure 1b shows percentages of participants with positive culture results, negative culture results, and no culture result.

Supplementary Figure 3a: Ziehl-Nielsen Smear Microscopy results by month of MDR-TB treatment and treatment success vs adverse outcome (n=362)

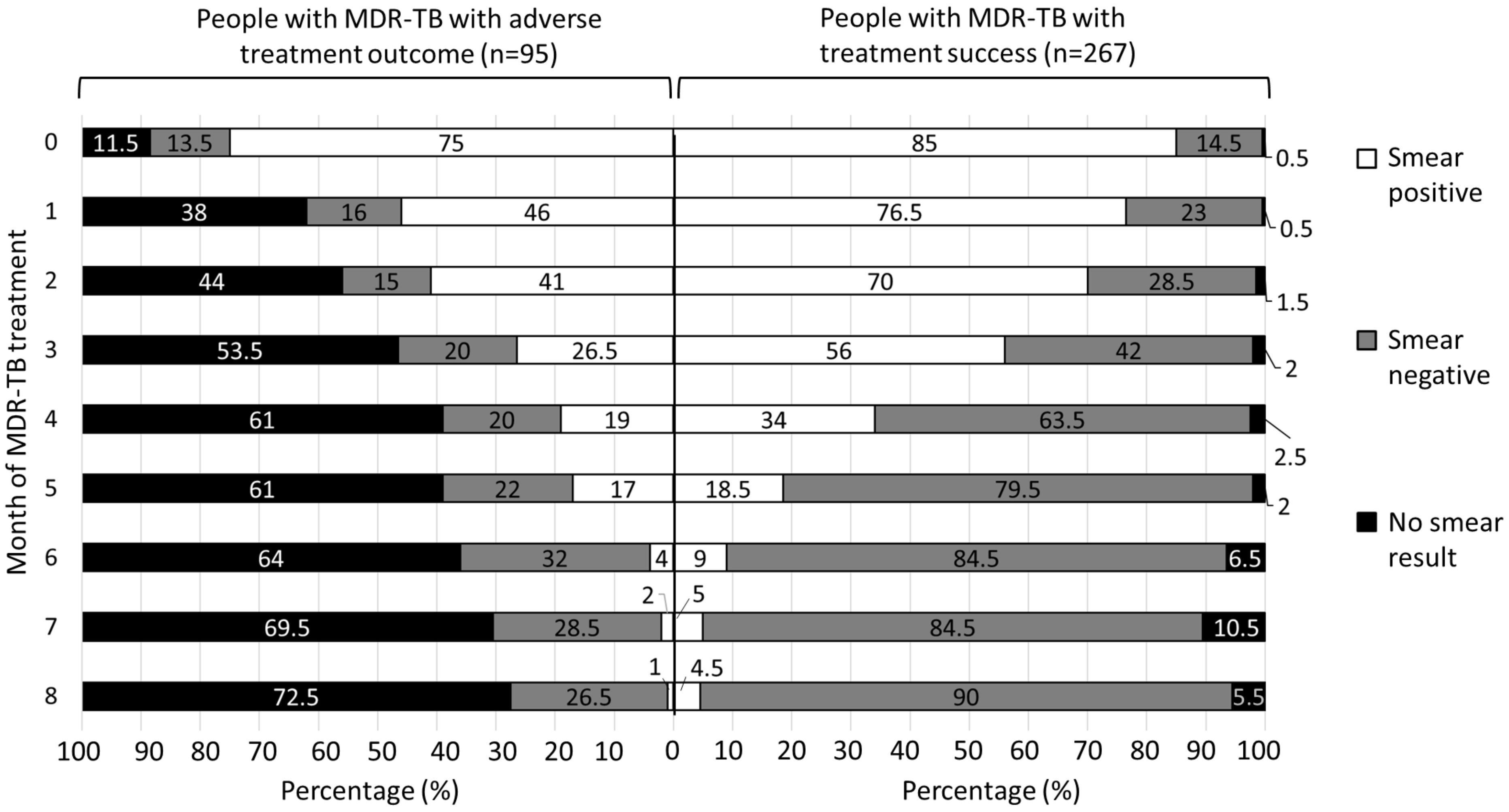

Supplementary Figure 3b: Mycobacterial culture results during MDR-TB treatment by treatment success vs adverse outcome (n=362)

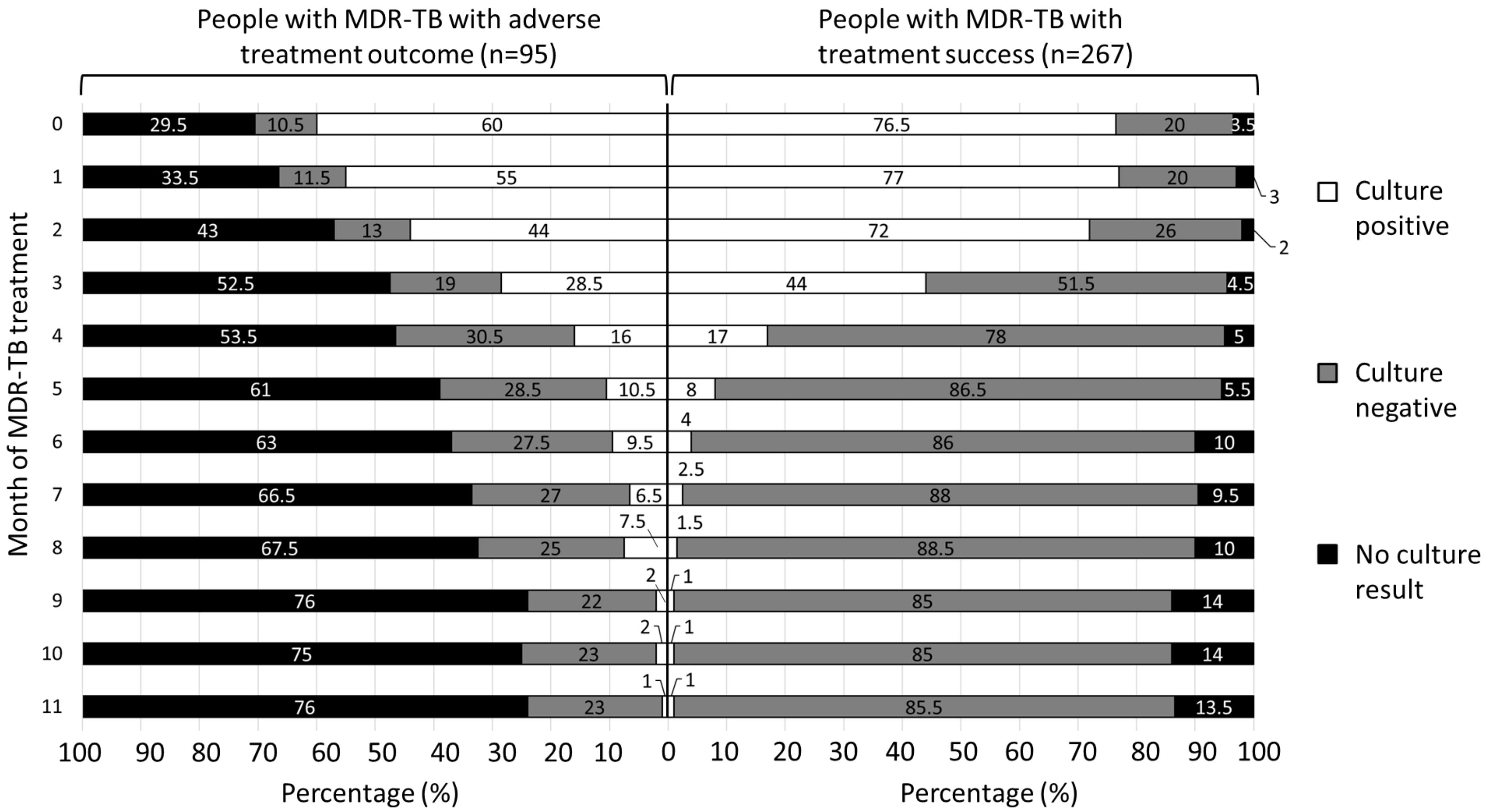

References

1. Dixit K, Rai B, Aryal T, Mishra G, Teixeira de Siqueira-Filha N, Paudel P, et al. Research protocol for a mixed-methods study to characterise and address the socioeconomic impact of accessing TB diagnosis and care in Nepal. Wellcome Open Res. 2020, 5:19 (https://doi.org/10.12688/wellcomeopenres.15677.2)

2. Rai B, Dixit K, Aryal TP, Mishra G, De Siqueira-Filha NT, Paudel PR, et al. Developing feasible, locally appropriate socioeconomic support for TB-affected households in Nepal. Trop Med Infect Dis. 2020;5(2):1–15.

3. World Health Organization. Definitions and reporting framework for tuberculosis - 2013 revision (updated Dec 2014 and Jan 2020) [Internet]. Vol. 18, Euro surveillance : bulletin Européen sur les maladies transmissibles = European communicable disease bulletin. 2020. 20455 p. Available from: http://www.ncbi.nlm.nih.gov/pubmed/23611033
